# Anti- *c-myc* cholesterol based lipoplexes as onco-nanotherapeutic agents *in vitro*

**DOI:** 10.12688/f1000research.25142.2

**Published:** 2021-01-07

**Authors:** Saffiya Habib, Aliscia Daniels, Mario Ariatti, Moganavelli Singh

**Affiliations:** 1 Department of Biochemistry, University of KwaZulu-Natal, Durban, KwaZulu-Natal, 4000, South Africa

**Keywords:** cancer, c-myc, siRNA, gene silencing, cationic liposomes

## Abstract

**Background:** Strategies aimed at inhibiting the expression of the *c-myc* oncogene could provide the basis for alternative cancer treatment. In this regard, silencing *c-myc* expression using small interfering RNA (siRNA) is an attractive option. However, the development of a clinically viable, siRNA-based, *c-myc* silencing system is largely dependent upon the design of an appropriate siRNA carrier that can be easily prepared. Nanostructures formed by the electrostatic association of siRNA and cationic lipid vesicles represent uncomplicated siRNA delivery systems.

**Methods:** This study has focused on cationic liposomes prepared with equimolar quantities of the cytofectin, N,N-dimethylaminopropylamido-succinylcholesteryl-formylhydrazide (MS09), and cholesterol (Chol) for the development of a simple, but effective anti- *c-myc* onco-nanotherapeutic agent. Liposomes formulated with dioleoylphosphatidylethanolamine (DOPE) in place of Chol as the co-lipid were included for comparative purposes.

**Results:** Liposomes successfully bound siRNA forming lipoplexes of less than 150 nm in size, which assumed bilamellar aggregrates. The liposome formulations were well tolerated in the human breast adenocarcinoma (MCF-7) and colon carcinoma (HT-29) cells, which overexpress *c-myc*. Lipoplexes directed against the *c-myc* transcript mediated a dramatic reduction in *c-myc* mRNA and protein levels. Moreover, oncogene knockdown and anti-cancer effects were superior to that of Lipofectamine™ 3000.

**Conclusion:** This anti- *c-myc* MS09:Chol lipoplex exemplifies a simple anticancer agent with enhanced *c-myc* gene silencing potential *in vitro*

## Introduction

Cancer is one of the leading causes of death world-wide. Deaths due to cancer have been reported to outnumber those due to acquired immune deficiency syndrome (AIDS), malaria and tuberculosis combined ^1^. Cancer treatment currently involves surgery, chemotherapy and/or radiation depending on the type and stage of the disease. Despite advances in understanding tumorigenesis and disease progression, these treatments are limited by harsh side-effects, the possibility of recurrences, and are heavily dependent on early detection and diagnosis for success ^2^. With the global cancer burden projected to increase to 21.7 million new cases and 13 million deaths by the year 2030, it is clear that more effective treatment strategies are required ^1^.

The altered activity of the *c-myc* proto-oncogene has been identified as an important element in the initiation and maintenance of the cancerous state of a cell. The *c-myc* gene encodes a nuclear phosphoprotein that is widely recognized for its role as a transcription factor. The c-Myc protein is believed to participate in the regulation of 10-15% of all genes ^3^. These include genes involved in cell-cycle progression, metabolism, cell growth, differentiation, adhesion, and apoptosis ^4– 10^. Hence, advances in the design of appropriate *c-myc*-silencing systems may prove useful in treating a broad range of cancers ^11^.

In theory, effective *c-myc* silencing may be achieved using endogenous cellular machinery, provided that the designed small interfering RNA (siRNA) molecule is successfully introduced. Several factors militate against the success of naked siRNA molecules *in vivo*. Naked siRNA molecules are highly susceptible to serum nucleases and are rapidly cleared by the kidneys ^11, 12^, while the size (~14 kDa) and negative charge of the siRNA prevents its passage across biological membranes ^13^. Therefore, an appropriate carrier is required to protect the siRNA from damage and elimination, as well as to disguise its negative charge. This system must have low toxicity, afford stability in serum, avoid recognition by the immune system, avoid renal clearance, and successfully deliver its contents to the RNAi machinery in the diseased cells.

Since nucleic acids can electrostatically associate with positively charged agents, a variety of cationic molecules as potential carrier vehicles have been investigated, cationic lipids receiving much attention, both in laboratory-scale experiments and clinical trials ^14– 16^. Cationic liposomes formed by the self-assembly of cationic and neutral or helper lipids, are the earliest cationic lipid-based delivery systems ^17, 18^. These form lipoplexes, when associated with siRNA. Their favorable characteristics such as safety, biocompatibility, and amenability to modification, have sustained their interest in cationic liposomal-siRNA delivery ^19^. Although several novel liposomal-siRNA systems have shown promise, none have resulted in a commercially available treatment ^16^. Major barriers to their application include poor stability in the bloodstream and early recognition by the immune system, as these positively charged lipoplexes associate with anionic serum proteins such as albumin and lipoproteins. This results in opsonization by serum components, destabilization of the lipoplex, and damage to the nucleic acid cargo before it reaches the diseased cells. Furthermore, lipoplexes often aggregate forming larger particles that accumulate in the lung and are rapidly cleared by the reticuloendothelial system, reducing dosage and circulation time ^20– 22^. Increase of the mechanical strength of the liposome bilayer to render it more resistant to the destabilizing action of serum proteins can be achieved by the incorporation of rigid, membrane-stabilizing lipids such as cholesterol (Chol) ^23, 24^.

This study involved the formulation of two cationic liposomes containing the cationic lipid N,N-dimethylaminopropylamido-succinylcholesteryl-formylhydrazide (MS09) combined with either the neutral helper lipid dioleoylphosphatidylethanolamine (DOPE) or the bilayer-stabilizing lipid, Chol. The transfection efficiency of these cationic liposomes on oncogenic *c-myc* expression at the mRNA and protein levels in the MCF-7 and HT-29 cell lines were evaluated.

## Methods

### Materials

DOPE, Chol, RIPA buffer, bicinchoninic acid (BCA) kit were obtained from Sigma-Aldrich (St. Louis, MO, USA). 2-[-(2-hydroxyethyl) piperazinyl]-ethanesulphonic acid (HEPES), ethidium bromide (10 mg/ml), tris(hydroxymethyl)-aminomethane hydrochloride (Tris HCl), 3-(4,5-dimethylthiazol-2-yl)-2,5-diphenyltetrazolium bromide (MTT), acridine orange (AO), ethylenediaminetetraacetic acid (EDTA), phosphate buffered saline tablets (PBS), sodium carbonate (Na _2_CO _3_) and sodium bicarbonate (NaHCO _3_) were supplied by Merck (Darmstadt, Germany). The siGENOME non-targeting siRNA #1 (D-001210-01-20), ON-TARGETplus SMARTpool Human MYC (4609) siRNA (L-003282-02-0020), 5× siRNA buffer (B-002000-UB-100, 0.3M KCl, 30mM HEPES, 1mM MgCl _2_, pH 7.5) and molecular grade RNase-free water (B-003000-WB-100) were purchased from Thermo Scientific Dharmacon Products (Lafayette, CO, USA). Ultrapure ^™^ agarose powder and Lipofectamine ^™^ 3000 reagent were procured from Invitrogen (Carlsbad, CA, USA). Sodium dodecylsulphate (SDS), iScript ^™^ genomic DNA (gDNA) clear complementary DNA (cDNA) Synthesis Kit (1725035), SsoAdvanced ^™^ Universal SYBR ^®^ Green Supermix (1725272), PrimePCR ^™^ SYBR ^®^ Green Assay: MYC, Human (Unique Assay ID: qHsaCID0012921); PrimePCR ^™^ SYBR ^®^ Green Assay: ACTB, Human (Unique Assay ID: qHsaCED0036269), PrimePCR ^™^ Template for SYBR ^®^ Green Assay: MYC, Human; PrimePCR ^™^ Template for SYBR ^®^ Green Assay: ACTB, Hard-Shell ^®^ 96-well PCR plates, 0.2 ml PCR tube strips, 10× Tris-buffered saline (TBS), 10% Tween 20, nonionic detergent; and blotting-grade blocker (non-fat dry milk), were supplied by Bio-Rad Laboratories (Richmond, CA). MCF 7 and HT-29 cells were obtained from the American Type Culture Collection (Manassas, VA, USA). Eagle’s Minimum Essential Medium (EMEM) with L-glutamine, Fetal bovine serum (FBS), trypsin-EDTA and penicillin/streptomycin mixture (10 000 U/ml penicillin, 10 000 μg/ml streptomycin) were purchased from Lonza BioWhittaker (Verviers, Belgium). All sterile plasticware were obtained from Corning Inc. (Corning, NY, USA). TRIzol ^®^ reagent (15596-026), c- *MYC* epitope tag antibody 9E11 (AHO0052), goat anti-mouse IgG2A secondary antibody, horseradish peroxidase (HRP) conjugate were obtained from Life Technologies (Carlsbad, CA, USA). β-actin antibody was purchased from Santa Cruz (Santa Cruz, CA, USA). Ultrapure 18 Mohm water was used throughout. All other reagents were of analytical grade.

### Synthesis of MS09 and liposome formulation

MS09 was prepared from cholesterylformylhydrazide hemisuccinate (MS08) via an active ester intermediate according to the method previously published ^23^. Briefly, the *N*-hydroxysuccinimide ester of cholesterylformylhydrazidehemisuccinate (0.083 mmol) and dimethylpropylamine (0.35 mmol) were dissolved in a H _2_O:pyridine:DMF (13:7:10, v/v/v) mixture. The product (MS09) was monitored and purified by thin layer chromatography. For liposome formulation, the lipids were combined in quantities as in Table 1 and concentrated to a thin film *in vacuo* (Büchi Rotavapor R rotary evaporator). The film was rehydrated (4°C, 48h) in sterile HEPES buffered saline (HBS, 20 mM HEPES, 150 mM NaCl, pH 7.5; 0.5 ml), vortexed and, then sonicated in a Transsonic (T460/H) bath-type sonicator (Singen, Germany). Liposome preparations were stored at 4°C and sonicated prior to use.

**Table 1.** Composition of prepared cationic liposomes.

Liposome	Lipid components (µmol)			Cytofectin concentration			Total lipid concentration		
MS09	DOPE	Chol	µmol/ml	µg/µl	mM	µmol/ml	µg/µl	mM	mM
MS09:DOPE	2	2	-	4	2.25	4	8	5.5	8
MS09:Chol	2	-	2	4	2.25	4	8	4.06	8

Chol = Cholesterol

### Liposomes and lipoplex characterization

The morphology of liposomes and siRNA lipoplexes were evaluated using cryogenic transmission electron microscopy (cryo-TEM), using a JEOL JEM.1010 transmission electron microscope (JEOL Ltd., Tokyo, Japan). Images were captured using an Olympus MegaView III digital camera in conjunction with SIS iTEM Universal Imaging Platform software (Shinjuku, Japan). Particle size and zeta (ζ) potential were determined using the Nanoparticle tracking analysis (NTA, NanoSight NS500, Malvern Instruments, Worcestershire, UK) fitted with a ZetaSight™ module and NTA 3.0 software.

### Gel retardation assay

Lipoplexes were prepared ( ^w^/ _w_) with varying amounts of the liposomes and a fixed quantity of siGENOME non-targeting siRNA (0.3 μg), as per Table 2. Lipoplexes (10 μl in HBS) were mixed with gel loading buffer (40% sucrose, 0.25% xylene cyanol, 0.25% bromophenol blue; 2.5 μl) and loaded onto 2% agarose gels. Electrophoresis was carried out for 30 min in a Mini-Sub ^®^ Cell GT electrophoresis cell (Bio Rad, Richmond, CA) containing tris-phosphate-EDTA (TPE) running buffer (36mM Tris-HCl, 30mM NaH _2_PO _4_, 10mM EDTA, pH 7.5) at 50 V. Gels were stained with ethidium bromide (0.1 μg/ml in water) for 30 min, and images were viewed and captured using a Vacutec Syngene G:Box gel documentation system, fitted with GeneSnap software, version 7.05.02 (Syngene, Cambridge, UK). Densitometric analysis was performed with the associated GeneTools software. The fluorescence intensities of unbound siRNA were expressed as a percentage against that of a naked siRNA control. The amount of liposome-associated siRNA at each MS09:siRNA ( ^w^/ _w_) ratio was determined as follows:

%boundsiRNA=100−%freesiRNA

**Table 2.** Varying amounts of MS09 liposomal formulations used in the gel retardation assay.

Liposome	MS09 liposome mass range (µg)						
MS09:DOPE	1.2	2.4	3.6	4.8	6	7.2	8.4
MS09:Chol	2.4	3.6	4.8	6	7.2	8.4	9.6

siRNA was kept constant at 0.3µg. Chol = Cholesterol

### Nuclease protection assays

Lipoplexes each containing 0.3 μg non-targeting siRNA were assembled at ( ^w^/ _w_) ratios of 12:1–32:1. These were incubated with 10% FBS at 37°C for 4h. A control, 0.3 μg naked siRNA was treated similarly. Nuclease activity was terminated using EDTA (10mM), and complexes destabilized with 0.5% ( ^w^/ _v_) SDS (55°C, 25 min). This was followed by electrophoresis as described previously.

### Cell viability assays

MCF-7 and HT-29 cells were seeded at densities of 4.0 × 10 ^4^ cells/well in 48 well plates, and maintained at 37°C for 24h, followed by treatment with lipoplexes containing non-targeting siRNA (14 nM) at ratios used in the nuclease protection assay. Controls with naked siRNA (14 nM) were included. Lipofectamine™ 3000 (LF3K), prepared according to the manufacturer’s protocol was used as a positive control. The LF3K transfecting complex (25 μl) had a final siRNA concentration of 25 nM. At 48h post-transfection, growth medium was aspirated, and cell viability was assessed by the MTT assay. Cells were incubated (37°C, 4h) with 200 μl medium containing 20 μl MTT (5 mg/ml in PBS) per well. Wells were then drained, and formazan crystals dissolved in DMSO (200 μl/well) to produce purple-colored solutions. Absorbance was read at 540 nm in a Mindray microplate reader, MR 96A (Vacutec, Hamburg, Germany), against a DMSO blank. Percentage cell viability was calculated as follows:

$$\frac{\left[{\text{A}}_{540\;\;\text{nm}}(\text{treated}\;\text{cells})-{\text{A}}_{540\;\text{nm}}(\text{blank})\right]}{\left[{\text{A}}_{540\;\;\text{nm}}(\text{untreated}\;\text{cells})-{\text{A}}_{\text{50}\;\;\text{nm}}(\text{blank})\right]}\times 100$$

### Gene expression assays

In all ensuing experiments, MS09:Chol and MS09:DOPE lipoplexes (MS09:siRNA ^w^/ _w_ = 16:1) contained 12 nM siRNA. MCF-7 and HT-29 cells were seeded in 6-well plates at densities of 3.0 ×10 ^5^ cells/well and incubated as previously described. Cells were transfected with lipoplexes as previously described. Lipoplexes were assembled with 12 nM of either non-targeting siRNA or ON TARGETplus SMARTpool Human *myc*-siRNA (anti-c- *myc-*siRNA). Transfections were carried out in triplicate, followed by cell harvesting for total RNA and protein.

### RNA extraction and RT-qPCR

Total cellular RNA was extracted using TRIzol ^®^ Reagent, and cDNA synthesis was carried out using the gDNA Clear cDNA Synthesis Kit, as per manufacturer’s instructions. For a single reaction, 0.8 μg of the RNA sample was used. Reaction mixtures were prepared on ice and the reactions were carried out in a C1000 Touch™ Thermal Cycler (Bio-Rad Laboratories (PTY) Ltd., Richmond, USA). The reaction was performed as follows: DNA digestion (25 °C, 5 min), DNase inactivation (75 °C, 5 min), and samples were maintained at 4 °C for 10 min. The RT supermix (4 μl) was added and cDNA synthesis was carried out as follows: priming (25 °C, 5 min), reverse transcription (46 °C, 20 min), RT inactivation (95 °C, 1 min) and samples were held at 4 °C for 10 min. Two cDNA synthesis reactions per RNA isolate were performed in parallel i.e. one reaction containing the RT supermix and a no RT control in which the no-RT supermix was added instead. cDNA samples were diluted to a final concentration of 25 ng/μl in nuclease-free water and stored at 4 °C, for no more than a week. The product of each cDNA synthesis reaction was subjected to RT-qPCR. A single reaction mixture (20 μl) contained SsoAdvanced™ Universal SYBR ^®^ Green Supermix (10 μl); primers (1 μl) specific for either the target gene, c-MYC (PrimePCR™ SYBR ^®^ Green Assay: MYC, Human) or reference gene, β-actin (PrimePCR™ SYBR ^®^ Green Assay: ACTB, Human); cDNA sample (25 ng, 1μl) and nuclease-free water (8 μl). Reaction mixtures in which DNA templates (either PrimePCR™ Template for SYBR ^®^ Green Assay: MYC, Human; or PrimePCR™ Template for SYBR ^®^ Green Assay: ACTB, Human; 1 μl) were substituted for cDNA served as positive controls. Reaction mixtures where nuclease-free water (1 μl) was substituted for either primers or cDNA were included as negative controls. All reactions were performed in triplicate. Reaction mixtures were prepared, on ice, in Hard-Shell ^®^ 96 well plates, sealed, briefly centrifuged and then loaded in a C1000 Touch™ Thermal Cycler (CFX 96 Touch™ Real-Time PCR Detection System, Bio-Rad Laboratories (PTY) Ltd., Richmond, USA). Data was analyzed with CFX Manager™ Software version 3.0, and *c-myc* expression was normalized to β-actin using the ΔΔCq comparative quantification algorithm.

### Total protein expression and ELISA

MCF-7 and HT-29 cells were seeded in 6-well plates at densities of 3.0 ×10 ^5^ cells/well and incubated as previously described. Cells were transfected with lipoplexes as previously described. The medium was removed, and cells washed twice with ice-cold PBS (1 ml/well). Cold (4°C) RIPA buffer (100 μl/well) was then added and cells placed on ice for 5 min with gentle shaking to dislodge cells. Cell suspensions were centrifuged (14 000 × g, 4°C, 15 min) and lysates/protein extracts were immobilized onto the wells of a 96-well plate with 50 mM carbonate-bicarbonate coating buffer (pH 9.6, at 4°C), overnight. Three replicates per isolate were performed. Each well received 10 μg protein in 100 μl coating buffer. The coating buffer was removed, and wells were rinsed twice with TBS (20 mM Tris-HCl, pH 7.5, 150 mM NaCl) containing 0.1% Tween 20 (TBST, 100 μl/well). Wells were then treated with 5% non-fat dry milk in TBST (100 μl) with gentle agitation to saturate unoccupied attachment sites. The blocking agent was removed, and wells rinsed twice with TBST (100 μl/well). Either c-myc (1:2000, in TBST) or β actin (1:10 000, in TBST) primary antibodies were added (100 μl/well) and incubated at room temperature for 1h. Primary antibodies were removed and wells washed with TBST (4x, 100 μl/well) for 5 min each, with agitation. The secondary antibody (1:2000, in TBST) was then added and incubated at room temperature for 1h. Wells were drained and washed with TBST, as previously. TMB (100 μl/well) was applied (room temperature, 30 min), and the reaction was terminated by the addition of 2M H _2_SO _4_ (100 μl/well). Absorbance was measured at 450 against a TMB (100 μl)/2M H _2_SO _4_ (100 μl) blank. Wells containing BSA (10 μg) served as negative controls. Antibody-free and substrate-free controls were included. Expression of c-myc was normalized to β actin and presented relative to untreated cells.

### Apoptosis assay

Live, apoptotic and necrotic cells were distinguished by the AO/ethidium bromide (EtBr) dual staining method ^25^. Lipoplexes were introduced to semi-confluent cells (8.0 ×10 ^4^ cells/well) in 24-well plates as previously described. After 48h, cells were rinsed with PBS (200 μl/well), stained with AO/EtBr solution (100 μg/ml AO and 100 μg/ml EtBr in PBS; 10 μl/well). Excess stain was removed by rinsing with PBS (100 μl/well). Cellular changes associated with apoptosis were observed under an inverted fluorescence microscope (CKX41, Olympus, Japan) at excitation and emission wavelengths of 540 nm and 580 nm, respectively. Images were acquired at 200× magnification using Analysis Five Software (Olympus Soft Imaging Solutions, Olympus, Japan). The % live/apoptotic/early apoptotic/late apoptotic/necrotic cells were calculated as below:

$$\frac{\text{[number}\;\text{of}\;\text{live/apoptotic/early}\;\text{apoptotic/late}\;\text{apoptotic/necrotic}\;\text{cells]}}{\text{[total}\;\text{cells}\;\text{counted]}}\times 100$$

### Statistical analysis

Statistical analyses were performed using one-way analysis of variance (ANOVA), followed by Tukey’s Multiple Comparison Test to compare between groups using GraphPad Prism version 5.04 (GraphPad Software Inc., USA). *P* values less than 0.05 were considered significant.

## Results

### Liposome and lipoplex characterization

**Figure 1.**
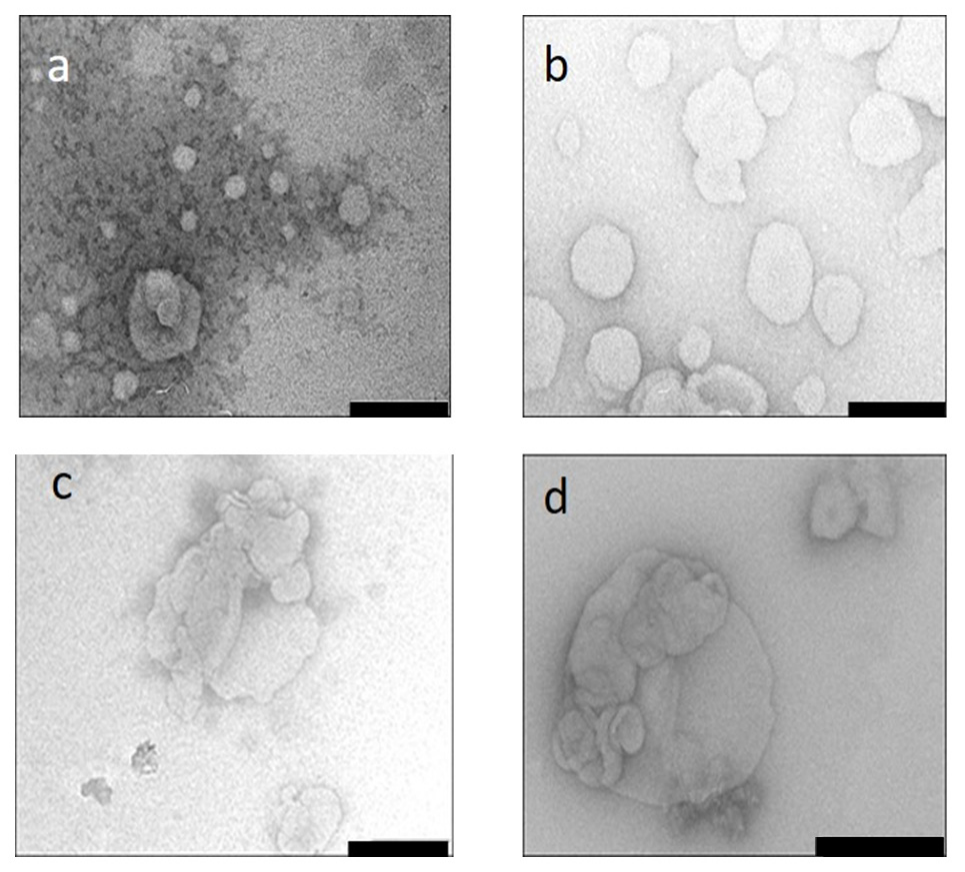
Transmission electron micrographs. Samples were viewed after uranyl acetate negative staining and flash freezing in liquid nitrogen. ( **a**) MS09:DOPE, ( **b**) MS09:Chol ( **c**) MS09:DOPE:siRNA ( **d**) MS09:Chol:siRNA. Bar = 200 nm

TEM presented MS09:DOPE and MS09:Chol formulations as round to irregular shaped unilamellar vesicles, respectively ( Figure 1a,b). The liposome-siRNA complexes ( Figure 1c,d) assumed structures that were different from the vesicles, emphasizing the heterogeneity and assembly of the liposome-siRNA complexes.

NTA results ( Table 3) show that MS09:DOPE and MS09:Chol liposome sizes were below 150 nm in size and may be classified as small unilamellar vesicles. The substitution of DOPE with Chol seems to have no significant effect on size or zeta potential, which remained high. It was observed that Chol is not likely to influence the electrical surface potential of liposomes because it does not bear an ionizable group ^26^. Lipoplexes were less than 150 nm in size ( Table 3), which is important for passive targeting of tumour cells via the enhanced permeability and retention effect.

**Table 3.** Particle sizes and zeta potentials of MS09 liposomal formulations and lipoplexes with siRNA at 16:1 ( ^w^/ _w_) ratios.

Liposome	Liposome		Lipoplex	
	Size (nm) ± SD	Zeta potential (mV) ± SD	Size (nm) ± SD	Zeta potential (mV) ± SD
MS09:DOPE	133.5 ± 15.2	-27.9 ± 5.4	92.4 ± 24.5	-33.6 ±4.5
MS09:Chol	137.5 ± 10.8	-26.9 ± 2.9	126.8 ± 7.3	-43.9 ±5.4

siRNA was kept constant at 0.3 µg. SD n=3; Chol= cholesterol

Zeta potential measurements are based on the interaction of the particle with ions in the medium in which it is dispersed. In this study, liposomes were dispersed in HEPES buffer, which can influence the zeta potential of a bilayer depending on the orientation of the molecule’s ionizable groups relative to the membrane ^27, 28^ in the electrical double layer. Hence, the negative zeta potential values do not necessarily imply that the liposomes would be unable to associate with siRNA molecules. Lipoplexes similarly displayed negative zeta potentials ( Table 3). Although it is accepted that the net positive charge of lipoplexes allows for binding to anionic membrane-associated proteoglycans to initiate cellular uptake ^29^, it is also possible for siRNA lipoplexes with negative zeta potential to enter cells and successfully facilitate gene silencing.

### Gel retardation assay

**Figure 2.**
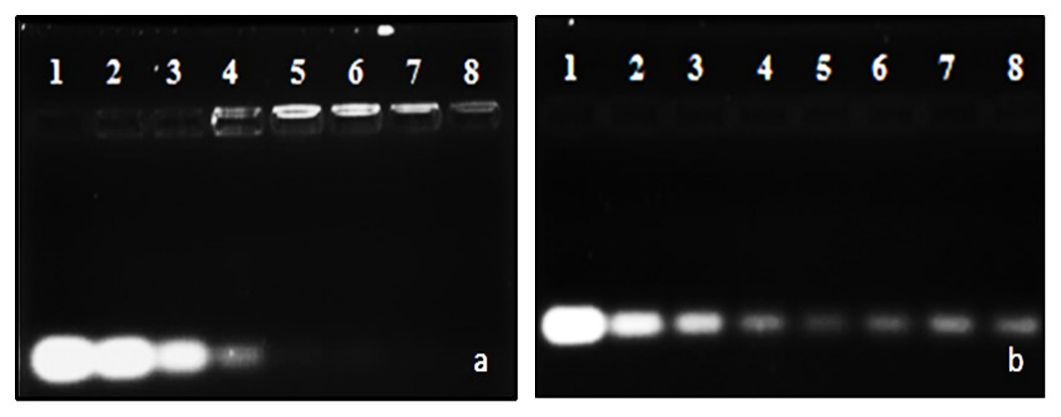
Gel retardation assay of the binding interactions between siRNA and cationic liposomes. Incubation mixtures (10 μl) contained siRNA (0.3 μg) and varying amounts of liposome corresponding with increasing amounts of cytofectin. Gels were viewed with ethidium bromide post-staining and UV _300_ transillumination. **a**) MS09:DOPE and **b**) MS09:Chol. In **a**) lane 1 contained naked siRNA and lanes 2-8, liposome-associated siRNA (4:1 – 28:1 ^w^/ _w_ ratios); and **b**) lane 1 contained naked siRNA and lanes 2-8, liposome-associated siRNA (8:1 – 32:1 ^w^/ _w_ ratios).

**Figure 3.**
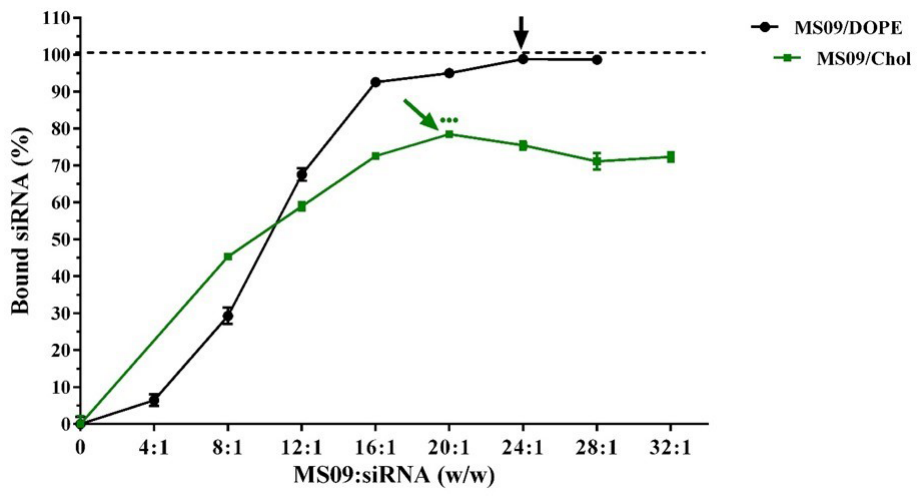
siRNA-binding at varying MS09:siRNA ( ^w^/ _w_) ratios, as determined by densitometry following gel retardation electrophoresis. Data is presented as the mean ± SD ( *n* = 3). The point at which each formulation best bound siRNA is indicated by an arrow. ^●●●^ *P* < 0.001 *vs.* DOPE-containing counterpart at the respective MS09:siRNA ( ^w^/ _w_) ratio at which maximum siRNA was bound.

The gel retardation assay or band shift assay is widely documented as the first step in assessing the siRNA-binding ability of cationic carriers ^30– 32^. This assay is based on the premise that the migration of siRNA is retarded in an electric field when bound to a carrier due to the formation of electroneutral complexes that are unable to permeate the gel matrix. MS09:DOPE was capable of fully preventing the migration of siRNA ( Figure 2a), whereas Figure 2b shows that although there was a decrease in unbound siRNA with increasing liposome content for the MS09:Chol formulation, unbound siRNA was evident at all MS09:siRNA ( ^w^/ _w_) ratios. Densitometric analysis ( Figure 3) confirmed that the binding of siRNA by these liposome formulations increased until a point, whereupon free siRNA in the gel persisted despite addition of liposome. This MS09:siRNA ( ^w^/ _w_) ratio was taken as the end-point/optimum binding ratio of these liposomes.

### Nuclease protection study

**Figure 4.**
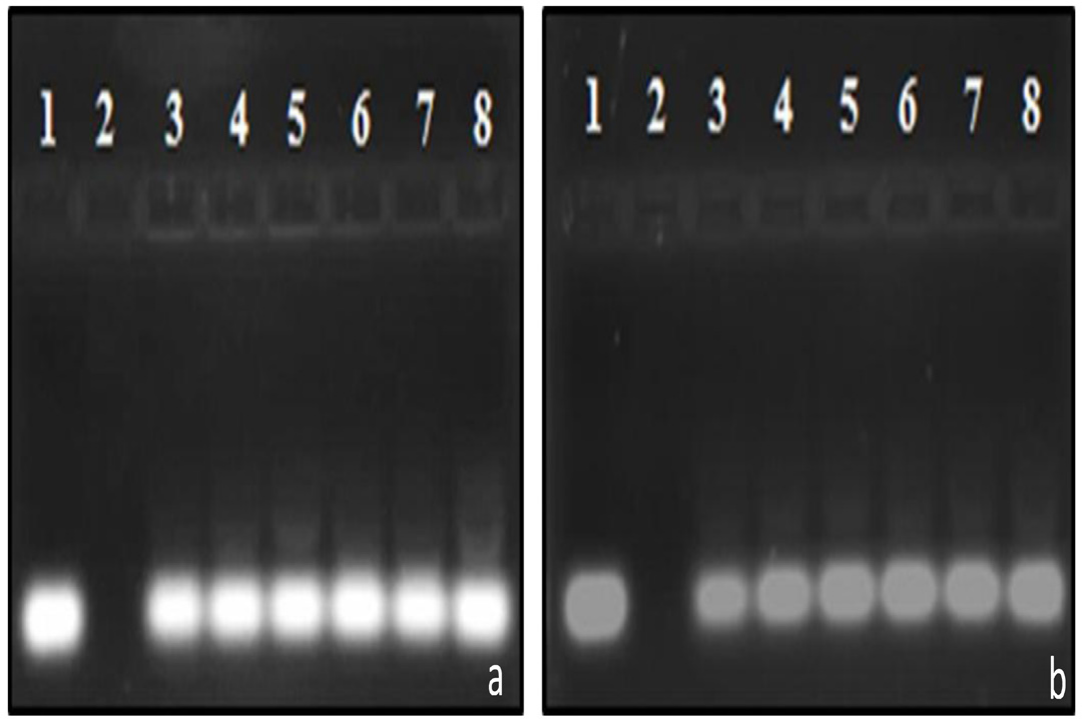
Nuclease protection capability of a) MS09/DOPE and b) MS09/Chol (1:1) liposomes in 10% fetal bovine serum for 4 h at 37 °C. In each gel, lane 1 contained undigested siRNA, lane 2, serum-digested siRNA and lanes 3–8, serum-exposed lipoplexes at varying MS09:siRNA (12:1 – 32:1 ^w^/ _w_) ratios.

**Figure 5.**
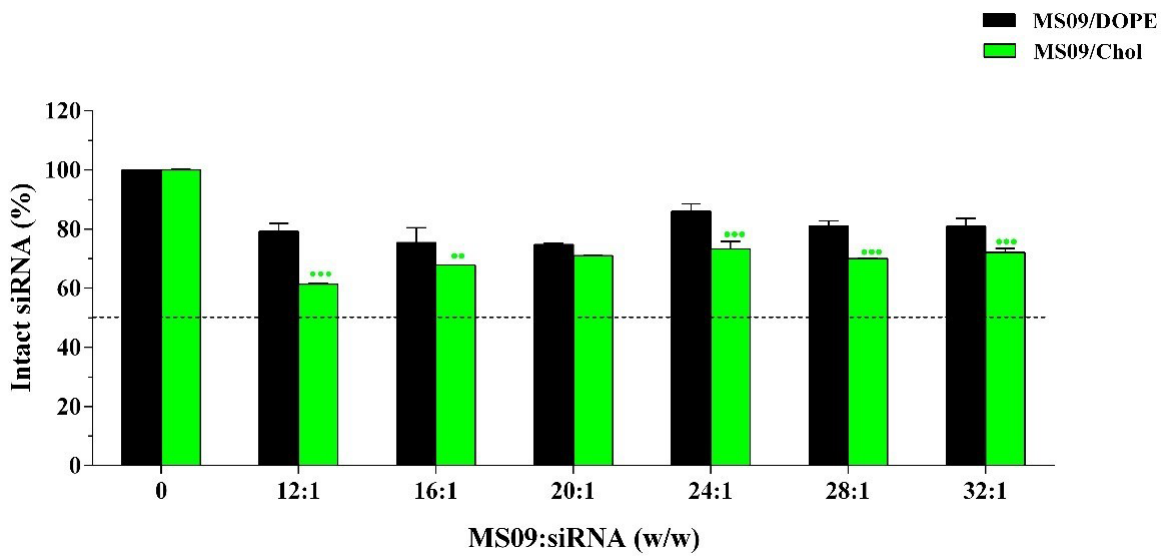
Densitometric analysis of siRNA-protecting capacity of liposomes in the presence of 10% fetal bovine serum. Intact siRNA associated with lipoplexes was quantified and expressed as a percentage of untreated siRNA (0.3 μg). Data is presented as the mean ± SD ( *n* = 3). ^●●^ *P* < 0.01, ^●●●^ *P* < 0.001 *vs*. DOPE-containing counterpart.

Attempts at lipid-mediated siRNA delivery are often frustrated by adverse interactions with serum. Figure 4 shows that, while naked siRNA was entirely degraded under experimental conditions (lanes 2), intact siRNA bands, less intense than the untreated control, were clearly visible in all instances. This shows that the liposomal formulations partially protected siRNA at the respective MS09:siRNA (w/w) ratios. Densitometric analysis of gels ( Figure 5) provided further insight into the siRNA-protecting capabilities of the liposomes. Maximum intact siRNA of 73% afforded by MS09:Chol liposomes at the MS09:siRNA ( ^w^/ _w_) ratio of 24:1 was significantly less than that of MS09:DOPE. 25% of siRNA was likely to be surface associated, as it was so loosely bound that it was coaxed off during electrophoresis. Such siRNA is readily detached from the liposomal bilayer upon exposure to serum nucleases ^33^.

### Cell viability studies

**Figure 6.**
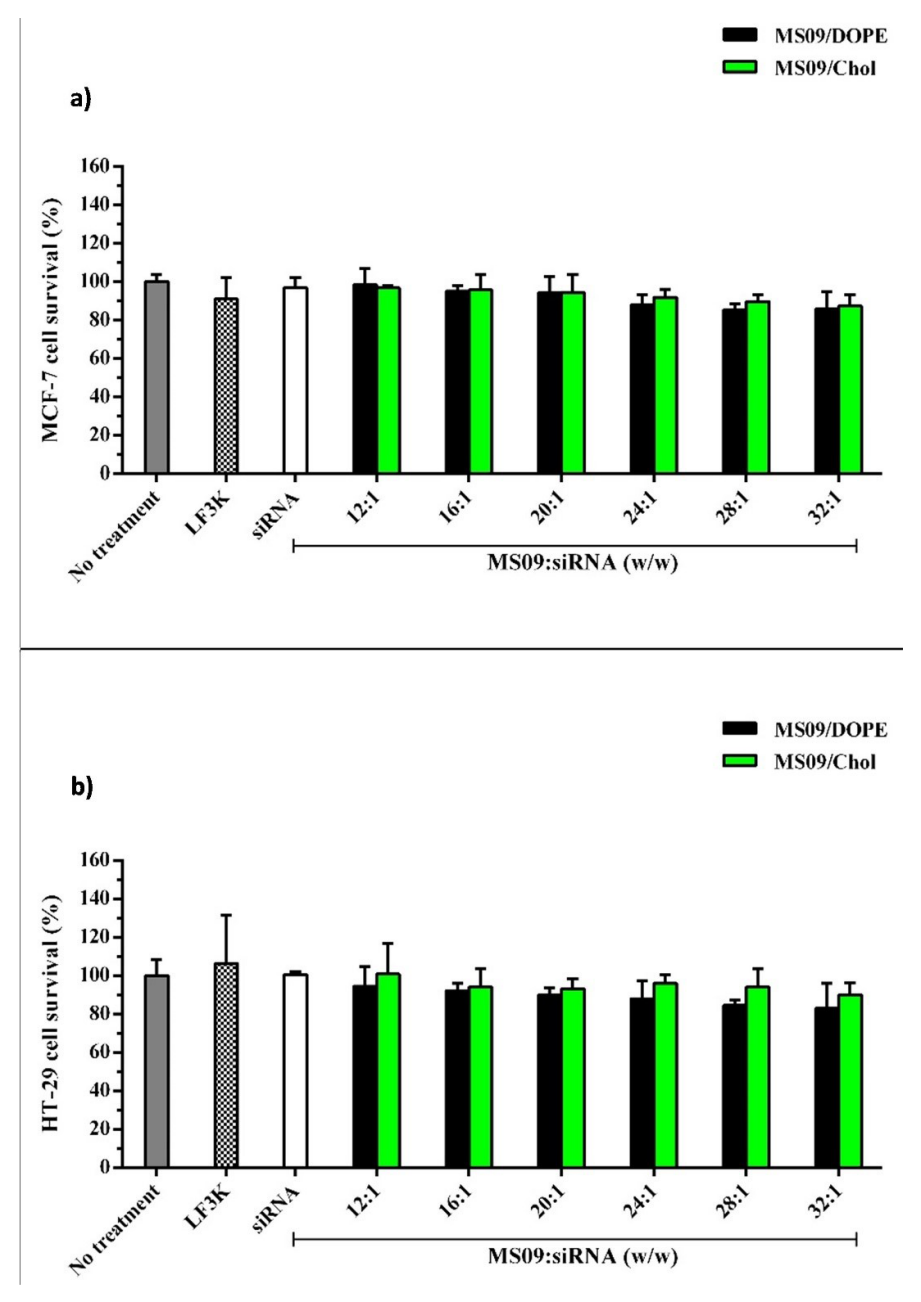
MTT viability assays with lipoplexes containing 14 nM siRNA. a) MCF 7 and b) HT-29 cells. Cells were exposed to lipoplexes for 48 h at 37 °C in the presence of serum. Each column represents the mean ± SD ( *n* = 3). *P* > 0.05 *vs.* untreated cells and DOPE containing counterparts. LF3K denotes Lipofectamine ^™^ 3000.

It was important that any growth inhibitory effects in the cancer cells be attributed solely to the delivered siRNA, and not due to any intrinsic harmful effect of the liposomal carrier. This assay is based on the principle that enzymes of the mitochondria of living cells reduce soluble MTT, a tetrazolium salt, to formazan crystals ^34^. The intensity of the resultant purple solution correlates with the extent of MTT reduction, and the number of viable cells ^35^. No significant reduction in viability was detected upon treatment of all cells with naked siRNA at concentrations contained in lipoplexes ( Figure 6). Cells retained viability of at least 88% with exposure to LF3K-siRNA complexes. In general, cell survival after exposure to the liposomal formulations were greater than 85% with no severe cytotoxicity evident.

### Gene silencing mediated by MS09:DOPE and MS09:Chol lipoplexes

**Figure 7.**
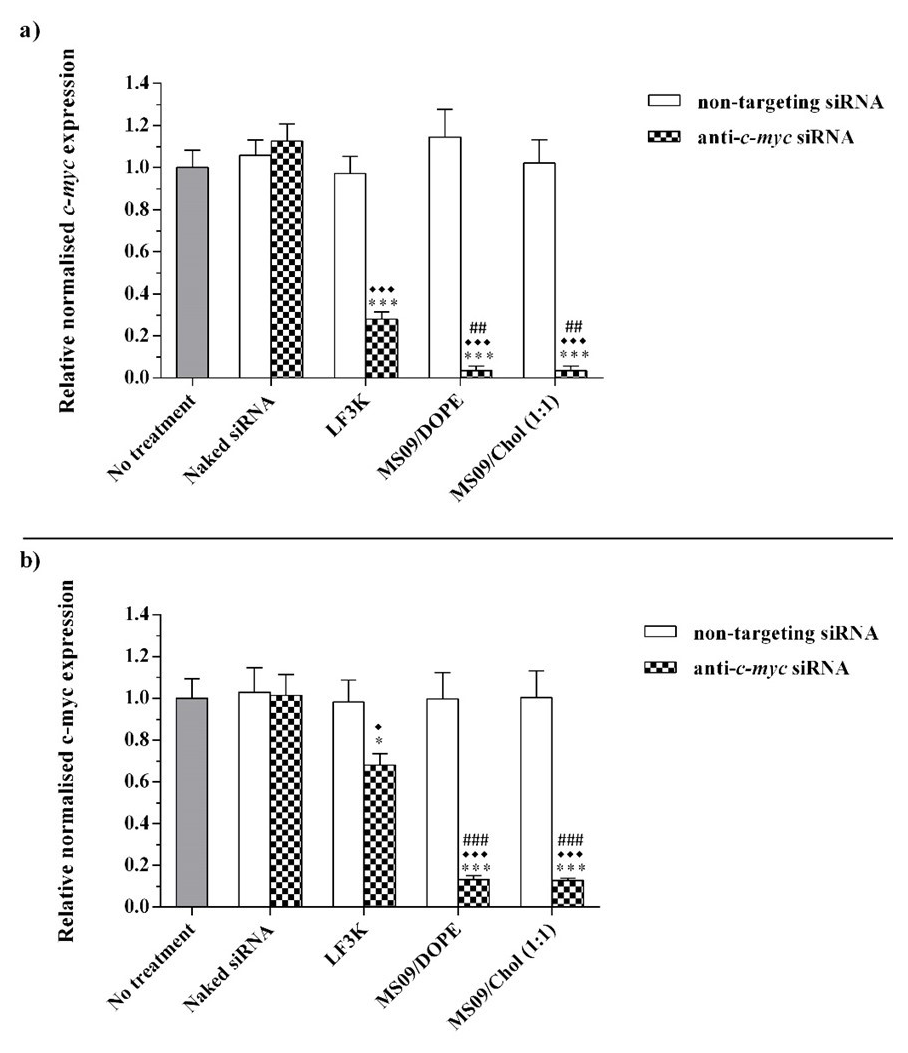
Effect of anti- *c-myc* lipoplexes on *c-myc*-mRNA expression in a) MCF-7 and b) HT-29 cells, following transfection with MS09:DOPE and MS09:Chol lipoplexes. *c-myc* expression was quantified by RT-qPCR and normalized to the *β-actin* reference gene. Each column represents the mean ± SD (n = 3). * *P* < 0.05, *** *P* < 0.001 *vs*. naked siRNA; ^♦^ *P* < 0.05, ^♦♦♦^ *P* < 0.001 *vs*. non-targeting siRNA; ^##^ *P* < 0.01, ^###^ *P* < 0.001 *vs*. anti *c-myc* LF3K. *P* > 0.05, with respect to anti *c-myc* MS09:Chol *vs*. anti- *c-myc* MS09:DOPE. LF3K= Lipofectamine ^™^ 3000.

**Figure 8.**
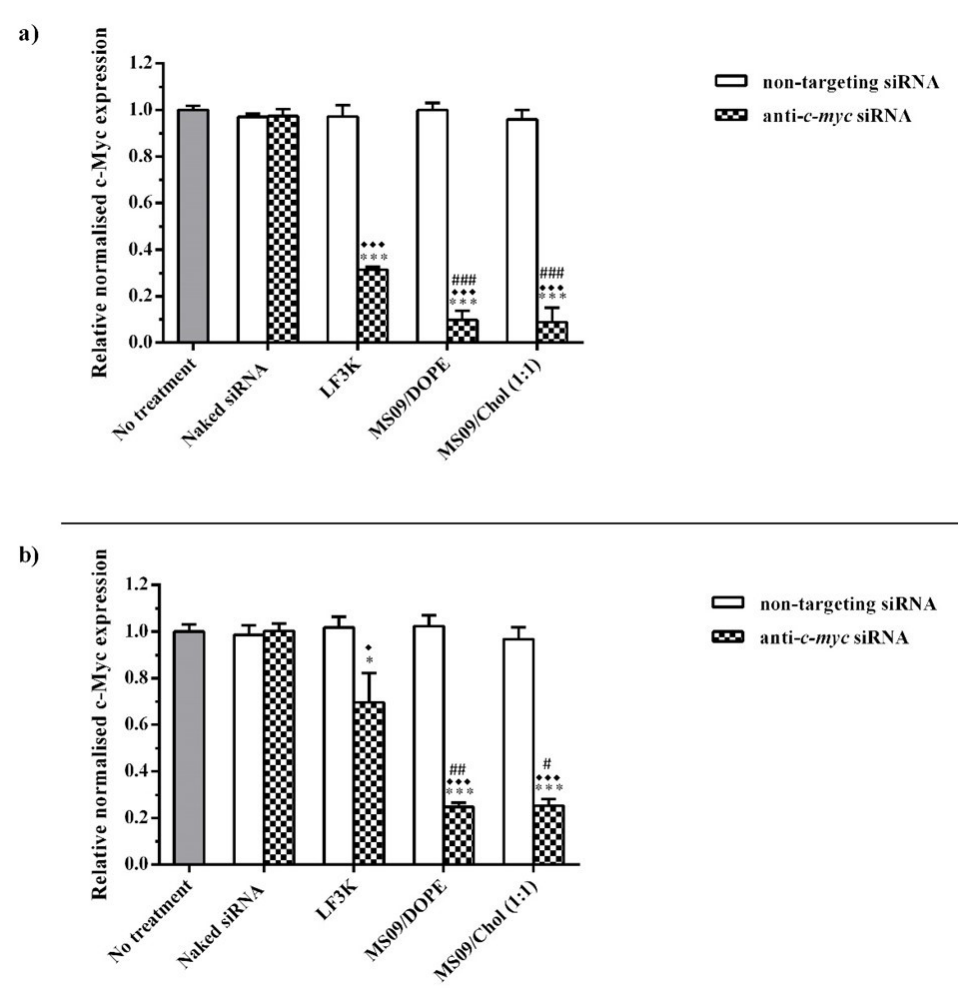
Effect of anti- *c-myc* lipoplexes on c-myc protein expression in a) MCF-7 and b) HT-29 cells, following transfection with MS09:DOPE and MS09:Chol lipoplexes. c-myc expression was quantified by ELISA, and normalized to the internal control, β-actin. Each column represents the mean ± SD ( *n* = 3). * *P* < 0.05, *** *P* < 0.001 *vs*. naked siRNA; ^♦^ *P* < 0.05, ^♦♦♦^ *P* < 0.001 *vs*. non-targeting siRNA; ^#^ *P* <0.05, ^##^ *P* < 0.01, ^###^ *P* < 0.001 *vs*. anti *c-myc* LF3K. *P* > 0.05, with respect to anti *c-myc* MS09:Chol *vs*. anti- *c-myc* MS09:DOPE. LF3K= Lipofectamine ^™^ 3000.

Given that the initial RNAi effect is exerted at the mRNA level, the effect of transfection with anti- *c-myc* lipoplexes on *c-myc* transcripts in cancer cells was studied using RT-qPCR. Figure 7 shows a decrease in *c-myc*-mRNA in instances where a transfecting agent was used to deliver anti- *c-myc*-siRNA sequences. Quantification of cellular c-myc protein by ELISA ( Figure 8) showed that a decrease in *c-myc* mRNA levels was, in all cases, accompanied by a concomitant reduction in protein expression. Complexes assembled with non-targeting siRNA were without effect. This confirmed that the observed reduction in *c-myc*-mRNA contributed to the RNAi effect of the delivered anti- *c-myc*-siRNA. The fact that naked anti- *c-myc*-siRNA did not influence *c-myc* expression in any way, highlights the need for a delivery vehicle.

MS09:Chol and MS09:DOPE lipoplexes produced significant gene silencing compared to LF3K in both cell lines. In the MCF-7 cell line, MS09 lipoplexes achieved 8- and 3.5-fold greater knockdown of *c-myc* than LF3K at the mRNA and protein levels, respectively. In HT-29 cells, the decrease in *c-myc*-mRNA and protein was 5- and 2.8-fold more for MS09 lipoplexes. The superior performance of MS09 lipoplexes is highlighted by the fact that these contained at half the final siRNA concentration as LF3K.

### Apoptosis assay

**Figure 9.**
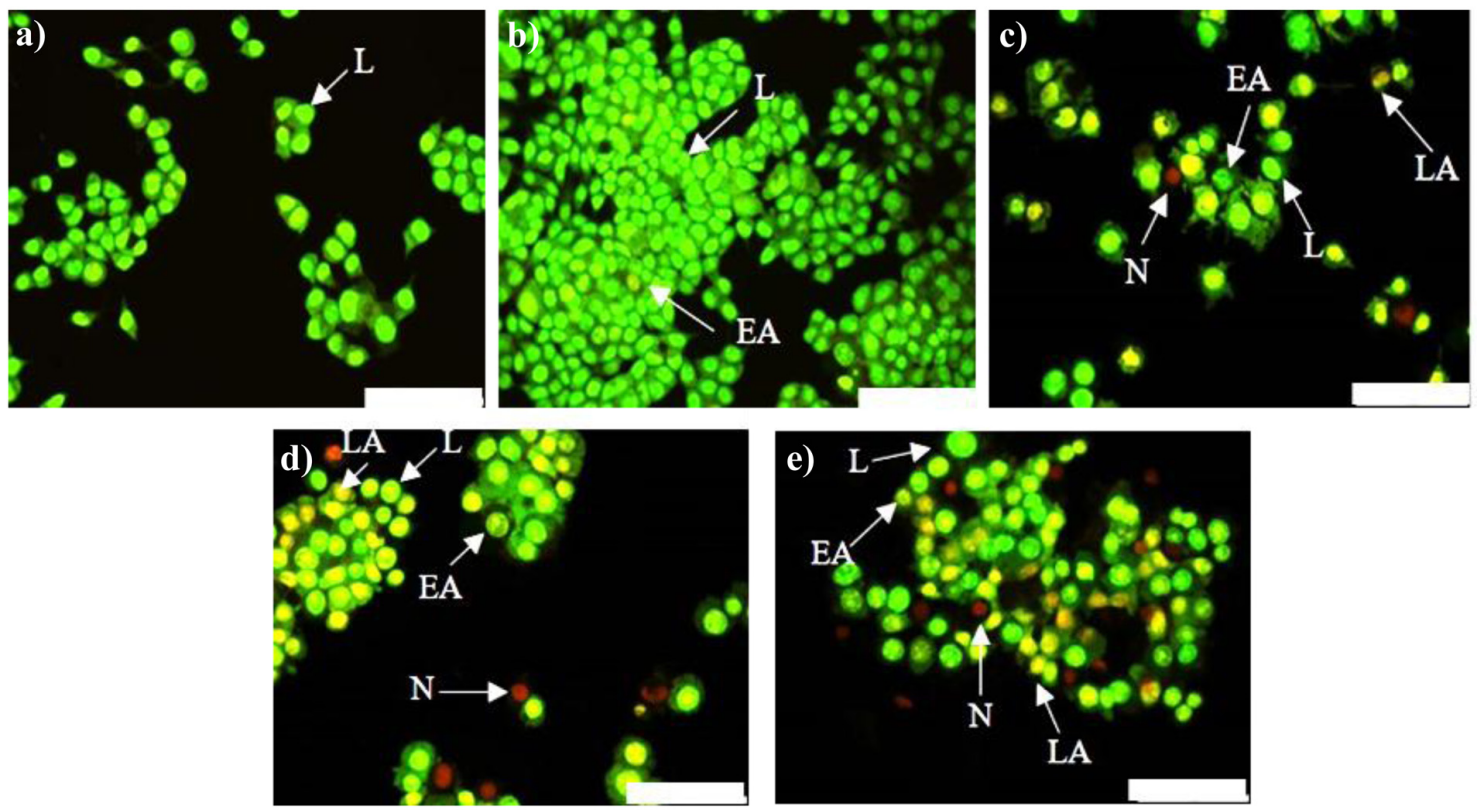
Apoptotic potential of anti- *c-myc* lipoplexes in MCF-7 cells. Cells were visualised 48 h post-transfection with AO/EtBr dual staining. **a**) Control, no treatment, ( **b**) naked anti- *c-myc*-siRNA, ( **c**) LF3K:siRNA, ( **d**) MS09:DOPE:siRNA and **e**) MS09:Chol:siRNA. Scale Bar = 100 μm. AO = Acridine Orange, EtBr = Ethidium Bromide, LF3K= Lipofectamine ^™^ 3000, L= live, EA = early apoptotic, LA = late apoptotic, N = necrotic.

**Figure 10.**
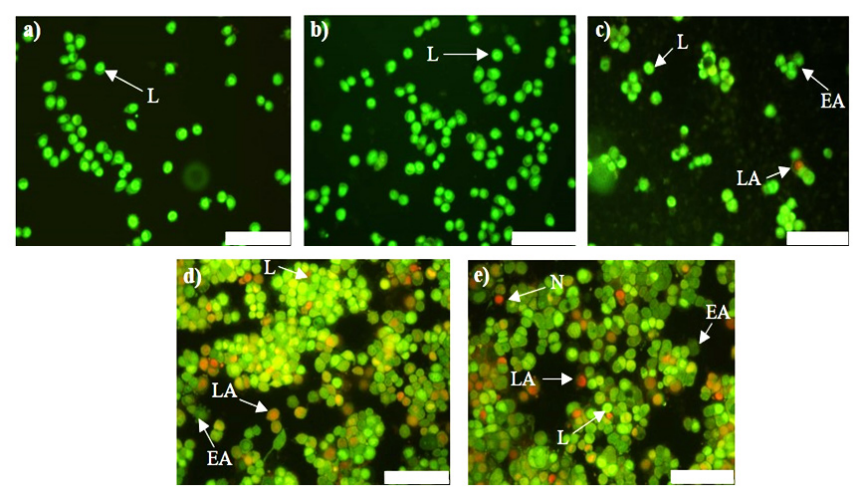
Apoptotic potential of anti- *c-myc* lipoplexes in HT-29 cells. Cells were visualised 48 h post-transfection with AO/EtBr dual staining. **a**) Control, no treatment, ( **b**) naked anti- *c-myc*-siRNA, ( **c**) LF3K:siRNA, ( **d**) MS09:DOPE:siRNA and **e**) MS09:Chol:siRNA. Scale Bar = 100 μm. AO = Acridine Orange, EtBr = Ethidium Bromide, LF3K= Lipofectamine ^™^ 3000, L= live, EA = early apoptotic, LA = late apoptotic, N = necrotic.

A desirable feature is for cancer treatment to induce cancer cell death without causing harm to surrounding healthy tissue. Hence, several anticancer approaches exploit the natural mechanisms of cell death such as apoptosis, that normally eliminates damaged and/or harmful cells in a regulated fashion ^36^. The AO/EtBr method is based on the principle that AO enters cells with intact plasma membranes and binds to DNA to emit green fluorescence, while EtBr enters cells with defective membrane integrity and fluoresces red-orange when bound to DNA. Differentiation between normal, early apoptotic, late apoptotic and necrotic cells was made based on observations of nuclear morphology ( Figure 9 and Figure 10). Live cells were characterized by a bright green nucleus in the center of the cell. The nuclei of early apoptotic cells, with undamaged membranes, also stained green, but appeared to be fragmented or condensed. In contrast, the nuclei of late apoptotic cells, with compromised membrane integrity, stained orange with evidence of fragmentation or condensation. Finally, necrotic cells were characterized by an intact bright orange nucleus ^37^.

The major mechanism of cell death observed with these anti- *c-myc* lipoplexes was apoptosis, which is similar to other studies demonstrating that inhibition of *c-myc* in cancer cells leads to apoptosis ^38– 40^. Importantly, necrosis, a non-specific form of cell death that is associated with an inflammatory response ^41^, was negligible in all instances, accounting for less than 3% of total cells per sample. Hence, MS09:Chol and MS09:DOPE-mediated anti- *c-myc-*siRNA delivery is capable of destroying cancer cells without damaging healthy tissue. The application of anti- *c-myc*-siRNA on its own did not result in any anticancer activity. Hence we can conclude that the anticancer effects can be ascribed to the RNAi activity of liposome bound anti- *c-myc*-siRNA.

## Discussion

MS09, a cationic lipid comprising three structural domains viz. a hydrophobic cholesteryl anchor and a polar dimethylammonium head group, separated by a 15Å spacer arm, was originally co-formulated with DOPE for the delivery of DNA ^23^ and siRNA ^42^ into mammalian cells. DOPE is a commonly used helper lipid in cationic liposome formulations ^43, 44^, but may not be suitable for intravenous administration. Early studies showed that the incorporation of Chol with phospholipids at 30 mol% or more resulted in the formation of a phase-separated region in the lipid bilayer ^45^. This property of Chol liposomes, in the absence of other helper lipids, becomes more pronounced and prevents adverse liposome-protein interactions, aggregation, improves mechanical strength and stability ^24, 45, 46^, and extending circulation time *in vivo* ^47^. It was reported recently that folate receptor-targeted cholesterol-rich liposomes readily form lipoplexes with Bmi1 siRNA that inhibit tumor growth both *in vitro* and *in vivo* ^48^. Furthermore, DNA in cholesterol-rich plasmid DNA lipoplexes was afforded full protection from enzymatic degradation and showed good efficacy in a CRISPR-Cas9 application in HEK293 cells ^49^.

TEM revealed that the liposomes were unilamellar, spherical vesicles that were capable of forming lipoplexes with the siRNA. Both the liposomes and lipoplexes were less than 150 nm in size, favoring targeting as nanoparticles of a suitable size will not pass through the tight junctions of normal blood vessels but can access tumor cells by passing through their more permeable vasculature and are retained because of reduced lymphatic drainage ^50^. These properties are valuable as determinants of lipoplex performance because they impact on the circulation time of lipoplexes in the body, accumulation at target sites, interaction with cells, the efficacy of cellular uptake and, gene silencing activity ^51^. Others have reported ligand-modification of anti- *c-myc* lipid-based nanoparticles ^40, 52^. While these may render delivery to tumour cells more effective and possibly alleviate short-term effects of *c-myc* inhibition in normal cells, it may not be an entirely necessary feature. This is with reference to findings that presented the feasibility of systemic *c-myc* inhibition with a non-discriminate *c-myc* inhibitor ^53^ and, that systemic administration of anti- *c-myc* siRNA in non-targeted liposomes did not inhibit the growth of cells with low *c-Myc* levels ^54^. Therefore, this study has focused on passive targeting of lipoplexes through optimising physical features to exploit the enhanced permeability and retention effect for effective intratumoural delivery of siRNA. In fact, preliminary experiments (not herein reported) show that the lipoplexes were not taken up by a non-transformed cell line. Moreover, a passive targeting strategy may pave the way for less elaborate anti- *c-myc* siRNA lipid nanoparticles that can be more easily and economically produced ^55^.

The negative zeta potential of the liposomes and lipoplexes did not affect their cellular uptake. It was reported that siRNA lipoplexes with negative zeta potentials were internalized by breast cancer cells via endocytosis and, that cellular uptake was dependent upon the activity of microtubules and actin ^56^. Negatively charged lipid-based siRNA nanocomplexes may be useful as they can avoid aggregation through interaction with erythrocytes and anionic proteins in biological fluids and have also been associated with lower toxicities than complexes carrying a net positive charge ^57, 58^.

MS09:DOPE and MS09:Chol liposomes showed good siRNA binding, but replacing DOPE with Chol at the same molar ratio in MS09 formulations weakened the siRNA interaction with MS09:Chol. It is possible that Chol may have induced arrangement of cytofectin molecules during vesicle formation such that a greater number of cationic centers were positioned inwards rather than on the surface of the bilayer. A further explanation may arise from the fact that Chol is a more rigid lipid than DOPE and results in liposomal bilayers that are less malleable. Therefore, during the process of lipoplex formation, the Chol-containing liposomes may not change conformation as easily as their DOPE-containing counterparts to completely encompass siRNA molecules. An early study with DOTAP/Chol liposomes, also demonstrated that Chol widened the interlamellar spaces of the resultant lipoplexes ^59^.

Both liposomes were capable of protecting the siRNA against nuclease degradation and were well tolerated by the MCF-7 and HT-29 cell lines. Cytotoxicity testing was performed under the same conditions as those employed in gene silencing experiments, except that lipoplexes were assembled with non-targeting siRNA so as to rule out the possibility of cell death due to silencing of any functional genes. In general, cell survival after exposure to the MS09:Chol formulation was better than its DOPE-containing counterpart. This could be attributed to Chol as an endogenous lipid, and that DOPE is pH-sensitive while Chol is not. This property of DOPE, which is regarded as important for transfection, has also been associated with toxicity, because it causes destabilization of lysosomes and release of debris within the cell ^60^.

Gene silencing experiments revealed that both MS09 liposomal formulations exhibited enhanced *c-myc* gene silencing in the MCF-7 and HT-29 cell lines, superior to that of LF3K. It was observed that *c-myc* inhibition at both levels of expression by all transfecting agents was more pronounced in the MCF-7 cells than in the HT-29 cells (P<0.05). This is underscored by the fact that HT-29 cells are considerably more difficult to transfect than other human cell lines ^61, 62^. This was also evident for the LF3K reagent with a markedly lower oncogene knockdown (1.4-fold reduction). In HT-29 cells cellular uptake of MS09:Chol and MS09:DOPE lipoplexes was comparable (P>0.05). The similar reduction in *c-myc-*mRNA and protein implied that the MS09 lipoplexes facilitated RISC-engagement of intact anti- *c-myc*-siRNA molecules with near-equal efficiency. However, in the MCF-7 cells, the MS09:Chol lipoplex, at 12 nM siRNA, facilitated effective siRNA delivery compared to DOPE-containing lipoplexes (P<0.05), but without a more pronounced gene silencing effect. This could be attributed to the possibility of saturation of the RNAi machinery, especially since this complex possibly releases siRNA directly into the cytoplasm. RISC saturation within a broad siRNA concentration range of 5-100 nM has been reported and is dependent upon the potency of the siRNA molecules involved ^42, 63^. Given the catalytic nature of siRNA activity, the results suggest that the MS09:Chol lipoplex could provide effective gene silencing at final siRNA concentrations below 12 nM in MCF-7 cells. For comparative evaluation as anti- *c-myc* agents, both MS09:Chol and MS09:DOPE lipoplexes were tested at the same MS09:siRNA (w/w) ratio, final lipid and siRNA concentration. Although the siRNA binding and protecting capability of MS09:Chol was shown to be weaker than its DOPE-containing counterpart at the MS09:siRNA (w/w) ratio of 16:1, the MS09:Chol lipoplex was proven to be as effective an siRNA carrier and *c-myc*-silencing agent. This could be attributed to the role of cholesterol nanodomains in transfection ^64^, and Chol-mediated fusion with the cell membrane as a mode of delivering intact siRNA directly to the RNAi apparatus in the cytoplasm ^65^.

A significant decrease in *c-myc* expression was correlated with anticancer effects following apoptosis analysis, which included inhibition of cancer cell migration, loss of cell viability and elimination of cancer cells through apoptosis, with the exception of anti- *c-myc*-LF3K in HT-29 cells. Here *c-myc* inhibition was too low to induce significant apoptosis and reduce cancer cell numbers. Overall, both anti- *c-myc* lipoplexes produced better anticancer activity than LF3K, in a given cell line. Furthermore, the comparable gene silencing activity mediated by MS09:Chol and MS09:DOPE lipoplexes was coupled with anticancer effects of near-equal potency. Although *c-myc* inhibition with MS09 lipoplexes was more pronounced (3.8- and 2.8-fold differences at the mRNA and protein levels, respectively) in MCF-7 cells than in HT-29 cells, their associated impact on cell migration, growth and apoptosis induction were similar. This could be due to the level of *c-myc* inhibition required to elicit anticancer activity differs among cell lines, or that more potent oncogene knockdown does not necessarily correspond with enhanced anticancer activity.

Overall, our study has shown that effective *c-myc* gene silencing was achieved with both MS09 lipoplexes. Gene silencing experiments with both liposomal formulations showed superior gene silencing compared to Lipofectamine™ 3000 *in vitro*. It is worth mentioning that preliminary cellular uptake experiments (not reported in this study) under physiological serum concentration (50% ^v^/ _v_) have highlighted the serum-resistance of the MS09:Chol lipoplexes. Taken together with the results reported, the new formulation is likely to be a good candidate for *in vivo* evaluation, so as to provide a clearer indication of its clinical applicability.

## Conclusion

The findings from this study show the potential of MS09-based siRNA cholesterol-rich lipoplexes for the effective silencing of the *c-myc* oncogene in the MCF-7 and HT-29 cells. This co-formulation of MS09 and Chol for siRNA delivery and its application in *c-myc* gene silencing was not previously explored but was shown to be an effective *c-myc* silencing agent with results comparable to its DOPE-containing counterpart. On a positive note, the fact that both MS09 lipoplexes have performed better in a recalcitrant cell line than the standard transfection reagent, confirms their applicability as oncogene silencing agents in difficult-to-transfect cancer cells, adding credence to their potential as broad-range anti- *c-myc* agents. Overall, these novel MS09:Chol liposomes are endowed with physicochemical properties that may render them more suitable for *in vivo* siRNA delivery than their MS09:DOPE counterparts, and that their anti- *c-myc*-siRNA lipoplexes should be developed further as a posttranscriptional intervention treatment modality for mono- and polygenic human diseases.

## Data availability

### Underlying data

Zenodo: Anti-c-myc cholesterol based lipoplexes as onco-nanotherapeutic agents in vitro, http://doi.org/10.5281/zenodo.3946640 ^66^.

This project contains the following underlying data:

- Uncropped/unedited electron microscopy images showing morphology of liposomes and siRNA lipoplexes
- Raw data for particle size and zeta potential of liposomes and siRNA lipoplexes
- Uncropped/unedited gel retardation assay electrophoresis images; and raw data for densitometric analysis of the gel retardation assays
- Uncropped/unedited nuclease protection assay electrophoresis images; and raw data for densitometric analysis of the nuclease protection assays
- Raw data for absorbance readings for MTT assays for MCF-7 and HT-29 cells
- Raw data for RT-qPCR quantifications for expression of *c-myc* and *β-actin*
- Raw data for absorbance readings for c-myc protein expression and b-actin using ELISA
- Uncropped/unedited apoptosis images; and raw data for percentage of live/apoptotic/early apoptotic/late apoptotic/necrotic cells calculated

### Extended data

Zenodo: Anti-c-myc cholesterol based lipoplexes as onco-nanotherapeutic agents in vitro, http://doi.org/10.5281/zenodo.3940426 ^66^.

This project contains the following extended data:

- Number of lipid molecules that constitute liposomal vesicles
- Size and size distribution of liposomes and lipoplexes by NTA summarized data
- Zeta potential and zeta potential distribution of liposomes and lipoplexes by Z-NTA summarized data
- Estimated number of liposomal vesicles and siRNA molecules per liposome-siRNA nanocomplex
- Flow profiles for liposome suspensions and lipoplexes
- Zeta potential and size vs. concentration graphs for liposome suspensions and lipolexes
- Miscellaneous calculations: estimation of the average number of lipid molecules per vesicle; N/P (+/) charge ratio; final siRNA concentration; final cytofectin and lipid concentration; estimation of average number of vesicles/nanocomplex; estimation of average number of siRNA molecules/nanocomplex
- Set-up for gel retardation and nuclease digestion assays
- Additional cytotoxicity data

Data are available under the terms of the Creative Commons Attribution 4.0 International license (CC-BY 4.0).
