# Supplementary material for: Anti- c-myc cholesterol based lipoplexes as onco-nanotherapeutic agents in vitro
Source: F1000Res. 2021 Jan 7;9:770. Originally published 2020 Jul 24. [Version 2] doi: 10.12688/f1000research.25142.2 (PMC7745184; doi:10.12688/f1000research.25142.2)
Supplement: Peer review history

# Peer review: Reviewer response for version 2

**DOI:** 10.5256/f1000research.44102.r76810

**Published:** 2021-01-08

**Authors:** Patrick Arbuthnot

**Affiliations:** 1 Wits-SAMRC Antiviral Gene Therapy Research Unit, Department of Molecular Medicine & Haematology, Faculty of Health Sciences, University of the Witwatersrand, Johannesburg, South Africa

The manuscript has been improved as a result of the revisions and is now suitable for indexing.

Is the work clearly and accurately presented and does it cite the current literature?

Yes

If applicable, is the statistical analysis and its interpretation appropriate?

Yes

Are all the source data underlying the results available to ensure full reproducibility?

Yes

Is the study design appropriate and is the work technically sound?

Yes

Are the conclusions drawn adequately supported by the results?

Yes

Are sufficient details of methods and analysis provided to allow replication by others?

Yes

Reviewer Expertise:

Gene therapy.

I confirm that I have read this submission and believe that I have an appropriate level of expertise to confirm that it is of an acceptable scientific standard.

# Peer review: Reviewer response for version 2

**DOI:** 10.5256/f1000research.44102.r76809

**Published:** 2021-01-07

**Authors:** Basiru Olaitan Ajiboye

**Affiliations:** 1 Department of Biochemistry, Phytomedicine and Molecular Toxicology Research Laboratory, College of Sciences, Afe Babalola University, Ado Ekiti, Nigeria

I will like to approve the manuscript for the indexing. Thanks so much.

Is the work clearly and accurately presented and does it cite the current literature?

Yes

If applicable, is the statistical analysis and its interpretation appropriate?

Yes

Are all the source data underlying the results available to ensure full reproducibility?

Yes

Is the study design appropriate and is the work technically sound?

Yes

Are the conclusions drawn adequately supported by the results?

Yes

Are sufficient details of methods and analysis provided to allow replication by others?

Yes

Reviewer Expertise:

Metabolic Diseases, Drug discovery, and Phytomedicine.

I confirm that I have read this submission and believe that I have an appropriate level of expertise to confirm that it is of an acceptable scientific standard.

# Peer review: Reviewer response for version 2

**DOI:** 10.5256/f1000research.44102.r76808

**Published:** 2021-01-07

**Authors:** Kaushik Pal

**Affiliations:** 1 Department of Physics and Astronomy, Iowa State University, Ames, IA, USA

I am fine with the present form of the report.

Is the work clearly and accurately presented and does it cite the current literature?

Partly

If applicable, is the statistical analysis and its interpretation appropriate?

Partly

Are all the source data underlying the results available to ensure full reproducibility?

Partly

Is the study design appropriate and is the work technically sound?

No

Are the conclusions drawn adequately supported by the results?

Partly

Are sufficient details of methods and analysis provided to allow replication by others?

Yes

Reviewer Expertise:

Chemistry, Chemical biology, biophysics, fluorescence microscopy

I confirm that I have read this submission and believe that I have an appropriate level of expertise to confirm that it is of an acceptable scientific standard.

# Peer review: Reviewer response for version 1

**DOI:** 10.5256/f1000research.27746.r67957

**Published:** 2020-12-16

**Authors:** Basiru Olaitan Ajiboye

**Affiliations:** 1 Department of Biochemistry, Phytomedicine and Molecular Toxicology Research Laboratory, College of Sciences, Afe Babalola University, Ado Ekiti, Nigeria

This manuscript successfully described how anti-c-myc cholesterol can be used as an onco-nanotherapeutic (in vitro). This research work will serve as an eye opener for possible management of cancer. As we all known that oncology  is a branch of medicine that deals with the prevention, diagnosis, and treatment of cancer, hence anti-c-myc cholesterol can be an example.

 Therefore, I want to use this medium to congratulate the authors and suggesting that the authors should carry out this experiment *in vivo* in future (if possible) so as to re-validate the *in vitro* results.

Is the work clearly and accurately presented and does it cite the current literature?

Yes

If applicable, is the statistical analysis and its interpretation appropriate?

Yes

Are all the source data underlying the results available to ensure full reproducibility?

Yes

Is the study design appropriate and is the work technically sound?

Yes

Are the conclusions drawn adequately supported by the results?

Yes

Are sufficient details of methods and analysis provided to allow replication by others?

Yes

Reviewer Expertise:

Metabolic Diseases, Drug discovery, and Phytomedicine.

I confirm that I have read this submission and believe that I have an appropriate level of expertise to confirm that it is of an acceptable scientific standard.

# Response

**Published:** 2020-12-31

**Authors:** Moganavelli Singh

**Affiliations:** University of KwaZulu-Natal, South Africa

We thank the reviewer for these kind and encouraging words

# Peer review: Reviewer response for version 1

**DOI:** 10.5256/f1000research.27746.r74490

**Published:** 2020-11-23

**Authors:** Kaushik Pal

**Affiliations:** 1 Department of Physics and Astronomy, Iowa State University, Ames, IA, USA

In this article, Saffiya Habib *et al*. presented the synthesis of Cholesterol, MS09 and DOPE-based composite liposome and applied that as the oncogene (c-myc) siRNA carrier. After synthesis, they have characterized the liposome with TEM, gel retardation assay, and checked efficacy with nuclease protection study, cell viability assay, gene silencing, and apoptosis assay. However, I feel this article can be only accepted after major revision. My specific comments are as follows:

1. The application of the liposome as the siRNA carrier is not new even from this very group this thing has been presented, so what is the advancement here? Is it the same platform with just a change in the siRNA? It needs to be clarified. Here my suggestion will be comparing the usefulness of the present and previously reported result for the same siRNA.
2. The Introduction of this article needs to be thoroughly revised. The research work done here and the introduction does not complement each other. The introduction is mostly talking about the *in vivo *challenges but the work is done *in vitro*.
3. In the Introduction, the authors claim that the main difficulty of this kind of carrier is the stability of itself and in the blood serum but there is no mechanistic clarification on how these liposomes are stable in solution and under serum spiked condition.
4. TEM image of the liposome is not clear and even in a small field of view the heterogeneity is not at all close to the state of the art ^1^. They should provide TEM images of the large field, size distribution (with the sampling number), etc.
5. The liposome after complexing with siRNA (lipoplex) looks like an aggregate structure rather than a single structure, this must be rectified.
6. In the Abstract author specify the liposome as bilamellar but in the Results section that stated/appears to be unilamellar. Also, the size said in the abstract is less than 200 nm which far away from the result. The author should be very objective in the abstract itself.
7. The comparison of the liposome is done with Lipofectamine™ 3000, which is not the commercial benchmark for the siRNA transfection. They should provide efficacy compare to the Lipofectamine™ RNAiMAX in all the cases.
8. Most of the figure captions are inadequate, they must be more informative.
9. The subfigures are not labeled, like Figure 2, it is very difficult to understand which well is loaded with what.

Overall, the whole manuscript is needed to be revised in order to make the Abstract, Introduction, Result, and Discussion to make completely to each other.

Is the work clearly and accurately presented and does it cite the current literature?

Partly

If applicable, is the statistical analysis and its interpretation appropriate?

Partly

Are all the source data underlying the results available to ensure full reproducibility?

Partly

Is the study design appropriate and is the work technically sound?

No

Are the conclusions drawn adequately supported by the results?

Partly

Are sufficient details of methods and analysis provided to allow replication by others?

Yes

Reviewer Expertise:

Chemistry, Chemical biology, biophysics, fluorescence microscopy

I confirm that I have read this submission and believe that I have an appropriate level of expertise to confirm that it is of an acceptable scientific standard, however I have significant reservations, as outlined above.

## References

[1] : Gene transfection efficiency of tracheal epithelial cells by DC-Chol–DOPE/DNA complexes. *Biochimica et Biophysica Acta (BBA) - Biomembranes*.1999;1419(2) : 10.1016/S0005-2736(99)00067-X 186-194 10.1016/S0005-2736(99)00067-X 10407070

# Response

**Published:** 2020-12-31

**Authors:** Moganavelli Singh

**Affiliations:** University of KwaZulu-Natal, South Africa

- The application of the liposome as the siRNA carrier is not new even from this very group this thing has been presented, so what is the advancement here? Is it the same platform with just a change in the siRNA? It needs to be clarified. Here my suggestion will be comparing the usefulness of the present and previously reported result for the same siRNA.

The use of liposomes as siRNA carriers has been previously reported. The novelty here lies in the formulation and its application. Firstly, the cytofectin MS09 has not previously been investigated in coformulation with cholesterol. Secondly, MS09 siRNA lipoplexes were previously investigated in serum-deficient conditions. Thirdly, the MS09:Chol:anti- *c-myc* siRNA system is introduced as an uncomplicated, easy-to-prepare *c-myc*-silencing, anti-cancer agent.

- The Introduction of this article needs to be thoroughly revised. The research work done here and the introduction does not complement each other. The introduction is mostly talking about the *in vivo *challenges but the work is done *in vitro*.

Although this study is exclusively *in vitro*, the *in vitro* experiments were performed as a first step towards preparing and developing a simple *c-myc* silencing lipid-based nanosystem that will ultimately be applicable *in vivo*. For this reason, considerations towards designing a clinically viable lipoplex were extensively outlined in the introduction as these provided a framework for the reported study.

- In the Introduction, the authors claim that the main difficulty of this kind of carrier is the stability of itself and in the blood serum but there is no mechanistic clarification on how these liposomes are stable in solution and under serum spiked condition.

All in vitro experiments were conducted under normal cell culture conditions, containing 10 % v/v serum. Generally, in vitro experiments are conducted in serum-free media which does not even come close to approximating in vivo conditions. Moreover, preliminary results obtained with  cellular uptake experiments of MS09:Chol lipoplexes in MCF-7 and HT-29 cells in 50 % v/v serum (physiological serum concentration) are encouraging.

- TEM image of the liposome is not clear and even in a small field of view the heterogeneity is not at all close to the state of the art ^1^. They should provide TEM images of the large field, size distribution (with the sampling number), etc.
- The liposome after complexing with siRNA (lipoplex) looks like an aggregate structure rather than a single structure, this must be rectified.

This has been corrected in the abstract.

- In the Abstract author specify the liposome as bilamellar but in the Results section that stated/appears to be unilamellar. Also, the size said in the abstract is less than 200 nm which far away from the result. The author should be very objective in the abstract itself.

The liposomes were unilamellar. However, the complexes of liposomes and siRNA (lipoplexes) to which reference is made in the abstract were bilamellar

- The comparison of the liposome is done with Lipofectamine™ 3000, which is not the commercial benchmark for the siRNA transfection. They should provide efficacy compare to the Lipofectamine™ RNAiMAX in all the cases.
- Most of the figure captions are inadequate, they must be more informative.

Additions to figure legends for Figure 1, 2, 4, 5, 6, 9 and 10 have been made.

- The subfigures are not labeled, like Figure 2, it is very difficult to understand which well is loaded with what.

Subfigures in Figure 2, 4 and 9 have been labelled.

# Peer review: Reviewer response for version 1

**DOI:** 10.5256/f1000research.27746.r67956

**Published:** 2020-08-06

**Authors:** Patrick Arbuthnot

**Affiliations:** 1 Wits-SAMRC Antiviral Gene Therapy Research Unit, Department of Molecular Medicine & Haematology, Faculty of Health Sciences, University of the Witwatersrand, Johannesburg, South Africa

The manuscript entitled ‘Anti- *c-myc* cholesterol based lipoplexes as onco-nanotherapeutic agents *in vitro*’ describes an analysis of non-viral lipid nanoparticles for the delivery of anti-c-myc siRNAs to cultured breast cells. The formulations comprising a cholesterol derivative termed MS09 in combination with DOPE or cholesterol performed significantly better than a commercially available transfection reagent. Useful properties of the complexes are described and extensive characterization is provided. The manuscript is written well, the study is neat and should be of interest to researchers in the field. Two points that should be addressed before indexing are the following:

1. Emphasis is placed on targeting cancer cells for delivery of an oncogene-disabling siRNA. The authors should provide information on how the vectors will/could be targeted to malignant cells specifically.
2. Ultimate utility of the vectors will be dependent on efficient action *in vivo*. Considerations for clinical translation of the technology should be provided.

Is the work clearly and accurately presented and does it cite the current literature?

Yes

If applicable, is the statistical analysis and its interpretation appropriate?

Yes

Are all the source data underlying the results available to ensure full reproducibility?

Yes

Is the study design appropriate and is the work technically sound?

Yes

Are the conclusions drawn adequately supported by the results?

Yes

Are sufficient details of methods and analysis provided to allow replication by others?

Yes

Reviewer Expertise:

Gene therapy.

I confirm that I have read this submission and believe that I have an appropriate level of expertise to confirm that it is of an acceptable scientific standard.

# Response

**Published:** 2020-12-31

**Authors:** Moganavelli Singh

**Affiliations:** University of KwaZulu-Natal, South Africa

- Emphasis is placed on targeting cancer cells for delivery of an oncogene-disabling siRNA. The authors should provide information on how the vectors will/could be targeted to malignant cells specifically.

The issue of tumour cell targeting has been included in the discussion: “ Others have reported ligand modification … … … more easily and economically produced”

  

- Ultimate utility of the vectors will be dependent on efficient action *in vivo*. Considerations for clinical translation of the technology should be provided.

The following has been included in the discussion: “It is worth mentioning that … … … a clearer indication of its clinical applicability.”
